# Surviving trisomy 18: A case report of a 5-year-old girl

**DOI:** 10.1097/MD.0000000000044225

**Published:** 2025-08-29

**Authors:** Mohamad A. Banat, Ramzi Mujahed, Sama S. Yaseen, Nada A. Makhalfeh, Shahed O. Rajabi, Baraa Abu Aisheh, Rama N. Basheer

**Affiliations:** a Faculty of Medicine, Palestine Polytechnic University, Hebron, West Bank, Palestine; b Pediatric Department, Princess Alia Governmental Hospital, Hebron, West Bank, Palestine.

**Keywords:** case report, congenital heart defects, Edwards syndrome, long-term survival, trisomy 18

## Abstract

**Rationale:**

Trisomy 18, often known as Edwards syndrome. It is a common chromosomal disorder characterized by the presence of an extra chromosome 18. Unfortunately, survival past the first year is quite rare, and there are only a few reports of individuals living long-term without needing corrective surgery. This case sheds light on an unusual situation where a patient survived for an extended period despite having severe congenital heart defects.

**Patient concerns:**

A 5-year-old girl, already diagnosed with trisomy 18, was admitted to the hospital after experiencing a cough and diarrhea that started after she began taking a nutritional powder supplement. The patient had a history of admission to neonatal intensive care for 1 month due to transient tachypnea of the newborn, mild retractions, and grunting. Additionally, she had intrauterine growth restrictions, dysmorphic features, and hypotonia.

**Diagnoses:**

Clinical examination revealed dysmorphic features, hypoxia, and a cardiac murmur. Chest radiography reveals central infiltration with cardiothoracic ratio 60%. Genetic testing confirmed the presence of trisomy 18, and an echocardiogram showed multiple congenital defects with significant right ventricular hypertrophy.

**Interventions:**

Initial management began with administering oxygen, performing metabolic tests, and a chest x-ray. However, because of ongoing low oxygen levels linked to her heart defects and pulmonary hypertension, long-term home oxygen therapy was initiated. A comprehensive supportive care with multidisciplinary team support was the main management.

**Outcomes:**

Throughout her treatment, oxygen saturation did not exceed 85%, and the patient’s development has remained severely delayed, with no significant motor or cognitive milestones. The patient had a long life expectancy for her complex heart defects, but eventually died of cardiac arrest.

**Lessons:**

This case shows the possibility of prolonged survival in trisomy 18, even with severe congenital heart defects, emphasizing the importance of multidisciplinary management and family-centered counseling. Documenting such cases expands understanding of this syndrome and guides long-term care strategies.

## 1. Introduction

Trisomy 18 syndrome, also known as Edwards syndrome, was first described by Edwards et al in 1960.^[1]^ It occurs in the full, mosaic, or partial trisomy 18q forms. It is the second most common autosomal trisomy after trisomy 21 (Down syndrome).^[2]^ Trisomy 18 is associated with serious and often fatal birth defects, resulting in a high mortality rate within the first month of life, with only 5% to 10% of live-born infants surviving beyond the first year.^[3,4]^ These birth defects commonly include cardiac anomalies, orofacial clefts, abdominal wall anomalies, tracheoesophageal anomalies, genitourinary anomalies, limb abnormalities, and nervous system anomalies.^[4]^ The prevalence of trisomy 18 ranges from 1 in 3600 to 1 in 10,000 live births, with an increased incidence observed over the past 2 decades, possibly due to rising maternal age. Moreover, while the prevalence is higher among females compared to males (3:2), fetal loss rates are higher in males, with females exhibiting better survival outcomes.^[5]^ This case report has been reported in line with the CARE (CAse REport) reporting guidelines.^[6]^

## 2. Case presentation

A 5-year-old girl with a known diagnosis of Edwards syndrome was brought to our emergency department. The patient was the fifth child of non-consanguineous parents. The mother was 42 years old at conception and had a history of smoking during pregnancy. The patient had 4 siblings, all without congenital anomalies. The pregnancy was unplanned and only discovered at the fifth month of pregnancy. Prenatal care was inadequate, with no folic acid supplementation, limited follow-up, and no detailed antenatal ultrasound.

She was delivered at 38 weeks of gestation via cesarean section because of breech presentation. Apgar scores were 7 at 1 minute and 8 at 5 minutes. Birth weight was 1900 g (<10th percentile) and head circumference 32 cm (10th to 50th percentile).

Shortly after birth, the patient was admitted to the neonatal intensive care unit for 1 month due to transient tachypnea, mild retractions, and grunting, receiving continuous positive airway pressure (continuous positive airway pressure with flow of 8 L/min, and positive end-expiratory pressure of 4 cm H₂O). She was referred from the obstetric hospital where she was born. Initial arterial blood gas analysis revealed the following: PH (7.3), PaCO_2_ (34 mm Hg), HCO_3_ (21 mEq/L), and PaO_2_ (85 mm Hg). Chest radiography revealed central infiltration with a cardiothoracic ratio 60%, demonstrating cardiomegaly. A neurologist was consulted due to hypotonia, and advised performing brain ultrasonography, which revealed a prominent posterior fossa. Given the presence of unexplained intrauterine growth restriction, dysmorphic features, and hypotonia, metabolic and genetic investigations were ordered, including thyroid function tests (normal), ammonia and lactate tests (normal), TORCH panel (normal), and karyotype tests. The patient received ampicillin and gentamicin for 2 days as empirical therapy for suspected sepsis; however, the septic culture results were negative. Therefore, the patient was diagnosed with low birth weight at term, intrauterine growth restriction, and transient tachypnea of the newborn. As the patient improved, she was discharged with oral feeding and was followed-up for karyotype analysis.

Cytogenetic analysis was performed using the G-banding technique, which revealed an abnormal female karyotype (47, XX, +18), with all the examined cells having trisomy 18 of the nondisjunction type. Results from the patient’s blood specimen for the study of chromosomal abnormalities were obtained via 72 hour stimulated cultures. All of the aforementioned interventions were performed in a pediatric referral center that serves as a primary institution for pediatric healthcare in the region.

Echocardiography performed on the first day of life revealed a large peri-membranous ventricular septal defect, patent ductus arteriosus (PDA), patent foramen ovale, and significant right ventricular hypertrophy. In addition, she had a history of pulmonary hypertension, quadriplegia, psychomotor impairments, and hearing loss. She had chronic severe protein-caloric malnutrition despite nutritional supplementation.

Psychomotor impairment was evident in the first month of life, likely secondary to chronic hypoxia due to heart defects and central hypotonia, which had been present since birth. The patient was unable to speak and could only communicate through nonverbal signals like grunting or smile. Also, she was unable to move independently or follow simple commands, completely dependent on basic daily activities, and unable to eat, walk, or talk. She experienced multiple episodes of oxygen desaturations at home, with oxygen saturation never exceeding 85% due to known congenital heart defects and pulmonary hypertension, necessitating 24-hours home oxygen therapy with a nasal cannula during her first 2 years.

From the first month of life, the patient has been under the care of multiple specialists including pediatricians, cardiologists, orthopedists, geneticists, and nutritionists. Despite interdisciplinary care, the patient’s development has remained severely delayed.

Pediatric cardiology follow-ups were conducted 3 times in her first year to check for cardiac defect progression, worsening, or improvement of the patient’s health status, and to assess the possibility of repairing the cardiac defect by invasive surgery. The prognosis for corrective heart surgery was poor because of the patient’s unclear future, as most trisomy 18 patients die at a very young age. Ultrasound did not reveal any renal or intestinal abnormalities.

There was no significant change in here cardiac condition after hospital discharge, except complaining of recurrent chest infections and oxygen desaturation, without any history of hospital admissions. She was treated conservatively at an outside clinic.

At 5-years of age, she was brought to our emergency department with a 7-day history of cough and episodes of diarrhea after starting nutritional powder supplementation by the family. No associated fever or vomiting was noted. There were no known surgical interventions according to the patient’s parents and no known drug or food allergies. No symptoms or signs of the other systems were observed. Her vaccinations were up to age.

**Figure 1.**
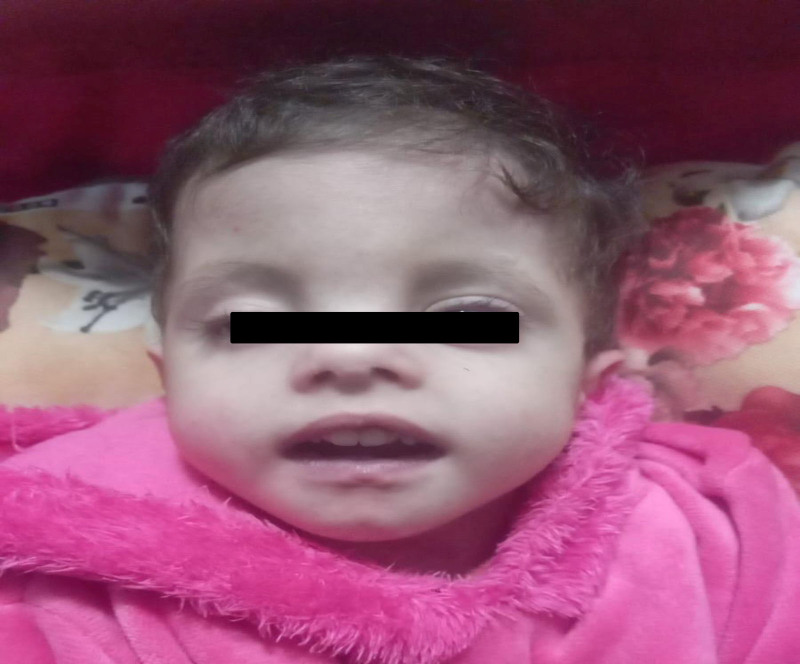
Facial picture showing dysmorphic features of Edward syndrome including: large forehead, low set ears, hypertelorism, unilateral ptosis, micrognathia, and upturned nose (The photos were taken with the consent of the patient’s parents.).

**Figure 2.**
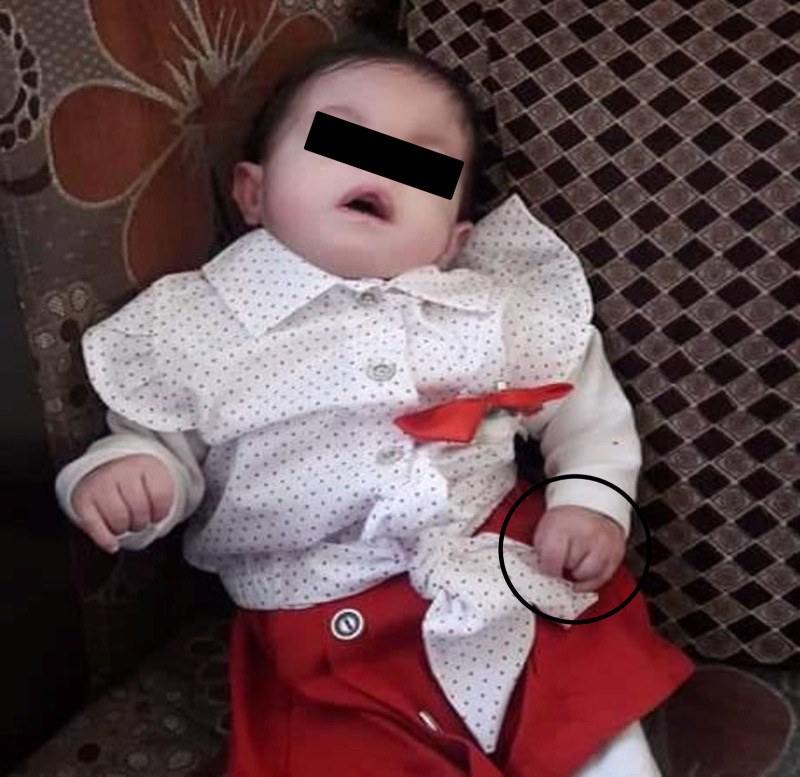
Clenched hand with overlapping of second and fifth fingers over the third and fourth figures, respectively (The photos were taken with the consent of the patient’s parents.).

**Figure 3.**
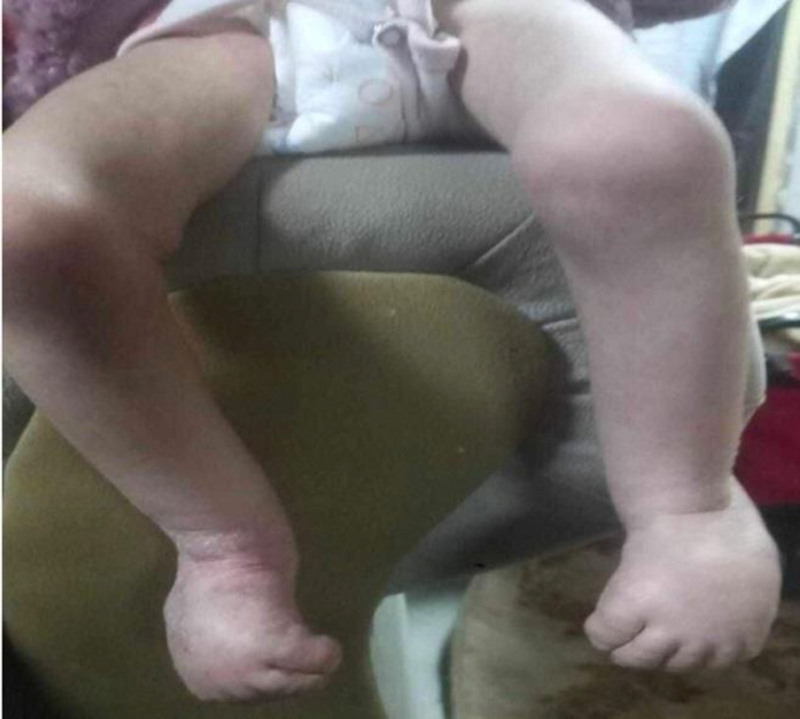
Bilateral clubfoot and curly toes combined with pronounced muscular atrophy (The photos were taken with the consent of the patient’s parents.).

Clinical examination revealed dysmorphic features consistent with trisomy 18, including a large forehead, unilateral ptosis of the right eye, hypertelorism, micrognathia, low-set ears, and upturned nose (Fig. 1). Physical features were observed such as overlapping of the second and fifth fingers over the third and fourth fingers, respectively (Fig. 2); bilateral clubfoot and curly toes (Fig. 3); and generalized hypotonia with retained reflexes. Chest auscultation revealed bilateral basal crackles, but no signs of respiratory distress. Cardiac auscultation revealed a grade 2/3 mid-systolic murmur. Abdominal examination revealed soft, lax abdomen without palpable organomegaly. Examination of other systems was unremarkable. Anthropometric measurements at her most recent assessment indicated a height of 60 cm and weight of 5 kg.

Laboratory tests revealed pancytopenia, with white blood cell count (3.4 × 10⁹/L), platelets (19 × 10⁹/L), and hemoglobin (9 g/dL). Given her presentation of recurrent chest infections, trisomy 18, and pancytopenia, the patient was admitted to the pediatric ward of the district general hospital for further evaluation and management. Sepsis was suspected but ultimately ruled out.

She was under medical supervision for long time and has experienced seizures for unclear reasons. The patient received conservative management, which included ongoing oxygen therapy, nutritional support, symptomatic treatment, and comprehensive multidisciplinary support. During the patient’s hospital stays and subsequent care, there were no recorded procedural complications or negative outcomes except of unexplained seizures. The patient died suddenly several months after discharge due to sudden cardiac arrest, as stated in the death report.

Diagnosis and continuous management face considerable obstacles due to financial constraints and insufficient access to prenatal care. The mother became aware of her pregnancy late (during the second trimester), did not receive folic acid or regular antenatal checkups, and was unable to pay for specialized assessments. These elements led to a postponed diagnosis and highlighted wider systemic challenges to care in low-resource environments.

The long-term prognosis for trisomy 18 is considered unfavorable, especially among those who also have intricate congenital heart anomalies. The majority of infants impacted by this condition do not live past their first birthday, and extended survival is extraordinarily uncommon. In this instance, the patient’s ability to reach the age of 5 years without undergoing surgery is viewed as remarkable and underscores the significant effect of reliable supportive care. The patients’ clinical data, including a diagnosis of trisomy 18, were retrospectively gathered from follow-up consultations conducted in 2024.

## 3. Discussion

Edwards syndrome is a genetic disorder associated with severe multisystem abnormalities. Approximately 90% to 95% of live-born infants do not survive beyond the first year of life, with a high intrauterine mortality rate.^[1,2]^ Females tend to have higher survival rates than males. These surviving patients often have severe psychomotor retardation and are primarily dependent on their caregivers.^[7]^ Survival to childhood is very rare, particularly in the absence of surgical repair of congenital defects.^[8]^

In this case, we describe a 5-year-old girl with classic trisomy 18 who survived well much longer than expected, despite having multiple cardiac defects and never undergoing corrective surgery for cardiac anomalies. This case is an example of the potential benefits of comprehensive supportive care.

Trisomy 18 has often been described as a case that is “incompatible with life.” However, there is increasing evidence that these patients can survive longer in life, especially with multidisciplinary team care and corrective surgical interventions. A recent scoping review by Schlosser et al in 2024 reported cases of liveborn infants with trisomy 18 survived beyond infancy, especially when some interventions, such as surgeries, were taken into consideration based on family preferences and clinical stability.^[9]^ A 16-year-old male with Edwards syndrome has been documented, further supporting the mounting evidence that some patients with trisomy 18 can live meaningfully long lives and that the condition is not always fatal. However, complex care is still required for these patients.^[10]^

Ninety percent of the patients with trisomy 18 have cardiac abnormalities. The condition is characterized by a number of common manifestations, including coarctation of the aorta, PDA, bicuspid aortic and pulmonary valves, atrial and ventricular septal abnormalities, and less frequently tetralogy of Fallot or transposition of the major arteries.^[1,2,11]^ In our case, the patient had ventricular septal defect, PDA, patent foramen ovale, and right ventricular hypertrophy, which commonly occur simultaneously and are all consider life-threatening without intervention. Recent studies found that surgical correction on individuals with trisomy 18 could increase life expectancy, lower the number of mortalities in the hospital, accelerate the rate of discharge, and improve survival rate.^[12,13]^ In this case, cardiac surgery was not recommended because of the overall poor prognosis, with the risks outweighing the potential benefits.

In our case, the family’s preference for supportive care, rather than aggressive intervention, was respected and resulted in unexpected longevity. However, our patient remains alive longer than expected despite having multiple congenital heart defects and not undergoing corrective surgeries. The patient’s survival can be attributed to careful home care, including continuous oxygen therapy and frequent oxygen saturation monitoring by caregivers.

To improve the survival rate of patients with Edwards syndrome, we urge that these cases be diagnosed early in pregnancy using noninvasive prenatal testing or amniocentesis. Recent research shows that chorionic villus sampling is a quick and accurate way to confirm high-risk noninvasive prenatal testing results for trisomy 21, 18, and 13. The study stressed that early confirmation through CVS allows for timely clinical decision-making, better parental counseling, better care planning, and earlier preparation for possible medical interventions or pregnancy management.^[14]^

Another notable finding in our case is the patient’s female sex and full-term birth, both factors associated with higher survival rate.^[15]^ There are many risk factors that may contribute to this condition, such as the mother’s age (42 years), mother’s smoking history, and lack of prenatal care, including not visiting a doctor prenatally, discovered pregnancy suddenly in the second trimester, and lack of folic acid and vitamin supplementation intake during pregnancy. Recent study reported by Pan et al highlights that advanced maternal age and female infants tend to survive longer,^[16]^ which might help explain this patient’s prolonged survival.

There has been a change in how trisomy 18 is perceived and treated, according to recent studies. At the age of 5, survival rates may rise to 23% with specific medical interventions. Parents generally report more favorable experiences, whereas physicians often perceive the child’s quality of life more negatively. These findings emphasize the importance of reassessing the blanket denial of treatment and advocate for personalized, ethically sound care strategies.^[17]^

Management should include a multidisciplinary team to provide a comprehensive treatment. For Edward’s patients with congenital cardiac abnormalities, early intervention with surgery or drugs such as diuretics and ACE inhibitors may increase survival. Supplemental oxygen or noninvasive ventilation (such as CPAP) can help with respiratory disorders, such as apnea and hypoxia. Recent study by Schlosser et al revealed that some children with this syndrome can live beyond infancy, with multidisciplinary support. But long-term outcomes remain guarded.^[9]^ Educating parents on the importance of regular follow-up visits to assess medical, developmental, and psychological progress, as well as providing counseling and emotional support to families, including information on end-of-life care if needed.

The authors promote shared decision-making and stress the significance of avoiding biases when counseling families. This method entails giving parent’s thorough information about all available care options, including comfort measures and medical and surgical procedures that are appropriate for the child’s condition. Ensuring fair, patient-centered care that upholds the family’s values and the child’s rights is the aim.^[18]^

## 4. Conclusion

Given the rarity of patients surviving beyond 1 year of life, particularly with severe and uncommon cardiac defects, we present the case of a 5-year-old female patient diagnosed with trisomy 18 and severe congenital heart disease. She also displayed characteristics such as plagiocephaly and hypotonia, which are rarely documented in the literature. A comprehensive supportive care, good clinical decision-making, and effective care strategies with multidisciplinary team support can improve survival in trisomy 18. To provide holistic care for these individuals, multiple medical specialists must be involved. Individualized counseling for parents should be provided, taking into account the patient’s condition and prognoses.

## Acknowledgments

The completion of this case could not have been possible without the participation and assistance of many people whose names may not be enumerated like our patient’s parents, their contribution are sincerely appreciated and gratefully.

## Author contributions

**Supervision:** Mohamad A. Banat, Ramzi Mujahed.

**Writing—original draft:** Mohamad A. Banat, Sama S. Yaseen, Nada A. Makhalfeh, Shahed O. Rajabi, Baraa Abu Aisheh, Rama N. Basheer.

**Writing—review & editing:** Mohamad A. Banat, Sama S. Yaseen, Nada A. Makhalfeh, Shahed O. Rajabi, Baraa Abu Aisheh, Rama N. Basheer.
